# Comprehensive genome analysis uncovers the diversity of jumbo phages in the pig gut microbiome

**DOI:** 10.3389/fvets.2025.1697229

**Published:** 2026-01-08

**Authors:** Chao Wei, Zhe Chen

**Affiliations:** National Key Laboratory of Pig Genetic Improvement and Germplasm Innovation, Jiangxi Agricultural University, Nanchang, China

**Keywords:** jumbo phages, phage diversity, pig guts, potential ecological interactions, potential novel jumbo phage families

## Abstract

Gut microbiome research has historically focused on bacterial communities. In contrast, the roles of viruses, especially jumbo phages, remain poorly understood. Jumbo phages are of major interest because their large genomes encode unique functions that can influence host metabolism and ecosystem dynamics. This study bridges this gap by identifying 1,545 jumbo phage genomes from 450 pig gut metagenomes. Using CRISPR spacer analysis, we predicted archaeal or bacterial hosts and reconstructed competitive phage networks within this ecosystem. Phylogenetic divergence combined with orthologous protein comparisons supported establishing 14 novel jumbo phage families. Functionally, 10 of these novel families encode auxiliary metabolic genes (AMGs) that enhance host metabolism alongside anti-defense systems including DNA methyltransferases, HNH endonucleases, and glycosyltransferases. Ecological interactions were further elucidated through co-abundance networks (*n* = 857 pairs) and CRISPR spacer matching (*n* = 425 pairs), revealing relationships between novel jumbo phages and other jumbo phages. Collectively, this work expands genomic resources for pig gut viromes and delivers new insights into jumbo phages’ functional capabilities, host associations, and global prevalence.

## 1 Introduction

Intestinal tract harbors a complex microbial ecosystem that includes bacteria, archaea, and viruses, all of which contribute to host health and ecological balance (1). Among these, bacteriophages (phages) are the most abundant members of the gut virome and play crucial roles by modulating bacterial communities through predation (2), horizontal gene transfer (HGT), and interactions with host immunity (3). Dysbiosis of this viral community has been linked to various diseases, highlighting its physiological importance (4).

A distinct and understudied component of the gut virome comprises jumbo phages, defined by their large genomes (>200 kb) (5, 6). These jumbo phages can encode complex functions, such as complete CRISPR-Cas systems, which may be used in host counter-defense or inter-viral competition (5, 7, 8). However, our understanding of jumbo phages remains limited due to challenges in obtaining their genomes from metagenomic data, leading to a sparse representation in current databases. This lack of genomic resources has hindered a systematic assessment of their diversity, distribution, and ecological roles across different environments, including animal guts (1, 9).

Pigs (*Sus scrofa*) serves as an important model for studying gut microbiome complexity (10, 11). While resources like the Pig Integrated Gene Catalog (PIGC) (12) and the Pig Virome Database (PVD) (13) have been developed, a dedicated genomic catalog of pig gut jumbo phages is still lacking. To address this gap, this study aimed to recover high-quality jumbo phage genomes from pig gut metagenomes. We performed comprehensive analyses on the resulting genomes to determine their taxonomy, predict hosts and lifestyles, identify novel phylogenetic groups, and assess their prevalence. Furthermore, we investigated phage-phage interaction networks through co-abundance analysis and CRISPR spacer matching. This work provides a foundational genomic resource and new insights into the biology of jumbo phages within the gut ecosystem.

## 2 Materials and methods

### 2.1 Recovering pig jumbo phage genomes using metagenomic sequencing data

Our one unpublished research collected 450 Fecal and gut content samples from eight countries, including China, Ghana, Denmark, France, Ireland, Canada, US, and Australia (Supplementary Table S1). All animal work was conducted according to the guidelines for the care and use of experimental animals established by the Ministry of Agriculture of China. The project was also approved by Animal Care and Use Committee (ACUC) in Jiangxi Agricultural University. Samples were processed for both bulk metagenomic sequencing and virus-like particle (VLP) enrichment. VLP-derived DNA was extracted using a ZR Viral DNA Kit, amplified via multiple displacement amplification, and sequenced on both Illumina (Novaseq, PE150) and PacBio (RS II, CCS mode) platforms. Bulk DNA was extracted with a QIAamp Fast DNA Stool Mini Kit and sequenced on Illumina. Raw reads were quality-controlled with fastp (v0.20.1) (14), followed by host DNA removal and *de novo* assembly using MEGAHIT (v1.2.9) (15) and Canu (16). Totally, we obtained 2,506,551 putative contigs to subsequent analysis. Viral contig identification employed a custom pipeline (Nayfach et al. (17)) where protein sequences were annotated against Pfam-A (18), TIGRFAM (19), and VOGDB^1^ databases using HMMER v3.3.2 (20) (hmmsearch-E 1e-5). Briefly, we identified viral contigs based on a combination of four viral signatures: (1) the presence of viral protein families, (2) the absence of microbial protein families, (3) the detection of viral nucleotide signatures, and (4) multiple adjacent genes on the same strand.

Phage identification utilized two complementary approaches. First, genomes required two or more genes containing virus-specific keywords [“capsid, phage, terminase, base plate, baseplate, prohead, virion, virus, viral, tape measure, tapemeasure neck, tail, head, bacteriophage, prophage, portal, DNA packaging, T4, p22, holin” (5)], exclusion of prokaryote-specific terms (“ribosomal protein, preprotein translocase, DNA gyrase subunit A”), and at least one spacer match from bacterial/archaeal genomes. Second, we applied PhaMer v1.0 (21) (Transformer-based phage predictor) with default parameters.

Identified phage genomes underwent quality control using CheckV v0.8.1 (22) for provirus boundary detection, host sequence removal, and completeness evaluation. Finally, genomes >200 kb were performed subsequent analysis.

### 2.2 Removing false positives of jumbo phage genomes

Given the inherent challenge in distinguishing viral and bacterial contigs due to horizontal gene transfer, we implemented rigorous false-positive removal. We evaluated bacterial gene content by quantifying hits against 318 bacterial universal single-copy orthologs (BUSCOs) (23). Protein sequences from putative jumbo phages were compared to BUSCO HMM profiles using hmmsearch (−E 0.05), with hits filtered using BUSCO-provided score cutoffs.

Following Gregory et al.’s methodology (24), we established a BUSCO ratio benchmark of 0–0.067 derived from Viral RefSeq genomes. Putative jumbo phage genomes exhibiting BUSCO ratios below this threshold (<0.067) were retained (*n* = 1,549), and 2 false-positive jumbo phage genomes were removed. Furthermore, we used hmmsearch (-T 30) to perform competitive searches against bacterial-specific, archaeal-specific, mixed, and viral-specific protein HMMs from the software VirSorter2 (25) for these retained jumbo phages. We enabled manual verification of viral-bacterial gene distributions, and removed 4 false-positive jumbo phage genomes. Finally, jumbo phages (*n* = 1,545) were categorized into the Pig Gut Jumbo Phage Genome Database (PGJPGD).

### 2.3 Lifestyle prediction and taxonomy assignment of jumbo phages

We predicted jumbo phage lifestyles using BACPHLIP v0.9.3 (26), which classifies genomes as virulent (score <0.5), uncertain (score 0.5–0.9), or temperate (score >0.9). Given their dual lytic-lysogenic capacity, we integrated CheckV-identified prophages with BACPHLIP-predicted temperate phages to define the final temperate category. Taxonomic assignment leveraged geNomad v1.7.4 (27), utilizing viral taxon markers aligned with most ICTV-recognized lineages.

### 2.4 Identifying alternative genetic codes in the PGJPGD

We employed Prodigal v2.50 (28) to identify open reading frames in all 1,545 pig gut jumbo phage genomes (PGJPGD) using four genetic coding schemes: the standard genetic code (11) alongside three alternative codes - TAG recoding (15), TAA recoding (90), and TGA recoding (91) (17, 29, 30). Jumbo phage genomes were assigned an alternative genetic code when their protein-coding density under code 15, 90, or 91 demonstrated a minimum 10% increase compared to density observed under standard code 11.

### 2.5 Identification of crAss-like phage in the PGJPGD

We identified crAss-like phages through sequential homology analysis (31, 32). First, all jumbo phage nucleotide sequences were queried against prototypical crAssphage proteins (UGP_018 polymerase and UGP_092 terminase; NC_024711.1) using BLASTx. Second, genome-wide nucleotide similarity was assessed via BLASTn against the p-crAssphage reference. Genomes exceeding 70 kb were classified as crAss-like phages if meeting either criterion: (1) BLASTx hits (E-value <1e-10) to polymerase or terminase, or (2) ≥95% nucleotide identity over ≥80% genome length with p-crAssphage.

### 2.6 Construction of phylogenetic trees for jumbo phage genomes of Caudoviricetes and crAss-like phages

We constructed phylogenomic trees for Caudoviricetes genomes following Low *et al*. (33). First, 77 gene markers were identified through HMMER searches against corresponding HMM profiles. Individual marker alignments were concatenated and trimmed using trimAl v1.4.rev22 (34), retaining fragments with <50% gaps. Genomes containing ≥3 markers present in >5% of alignment columns were retained. Final trees were reconstructed with IQ-TREE2 v2.1.3 (35) (1,000 bootstrap iterations) and visualized in iTOL^2^ (36).

For crAss-like phages, we generated TerL protein trees using established methods. TerL sequences were aligned with MAFFT (−-localpair --maxiterate 1,000), trimmed via trimAl with default parameters, and processed with IQ-TREE2. Resulting phylogenies were visualized in iTOL.

### 2.7 Host prediction for jumbo phages and identification of phage-phage interactions

We predicted hosts for pig gut jumbo phages using iPHoP v1.3.3 (37), which integrates multiple prediction methodologies. To identify potential interactions between phages, we analyzed CRISPR spacers within jumbo phage genomes, which represent a genetic memory of prior encounters with other mobile genetic elements. All genomes were processed with MinCED (38) with default parameters to identify CRISPR spacer arrays, followed by reciprocal blastn searches against identified spacers. Interactions were confirmed when viral spacers mapped to viral genomes with ≤1 mismatch and ≥95% coverage.

We further characterized defense systems through three complementary approaches: (1) CRISPR-Cas systems were predicted in jumbo phages and hosts using CRISPRCasFinder (-mNS 2 option). (2) Anti-CRISPR proteins (Acrs) were identified with Acafinder (39) (−l 800 -i 300 -b 10 parameters). (3) Host defense systems were profiled using DefenseFinder (40) with default settings.

### 2.8 Gene-sharing network analysis for pig gut jumbo phage genomes

We constructed a gene-sharing network using vCONTACT v2.0 (41), incorporating pig gut jumbo phage genomes alongside prokaryotic viruses from NCBI RefSeq (release 211). Network visualization employed Cytoscape v3.7.1 (42) with edge-weighted spring-embedded layout following established methodology (43).

Complementary phylogenomic analysis utilized the ViPTree web server v4.0,^3^ generating whole-proteome maximum-likelihood trees for complete jumbo phage genomes and related references. This analysis was supported by orthologous fraction calculations through CompareM (-evalue 1e-5 -identity 30%),^4^ quantifying shared proteins between genomes.

For the identification of potential novel family-level taxa, we applied a dual demarcation criterion consistent with operational standards for the class Caudoviricetes as endorsed by the International Committee on Taxonomy of Viruses (ICTV, e.g., ICTV Taxonomy Proposals 2022.003A. A) and some robust phylogenomic studies (43, 44). Specifically, genomes were proposed to belong to distinct novel potential families if they met both of the following thresholds in comparative analyses: (1) Phylogenetic Divergence: They resided on distinct branches separated by an evolutionary distance (log-scale branch length) of ≥ 0.05 in the whole-proteome tree. (2) Genomic Distinctness: They shared < 10% of their orthologous protein clusters (OPCs) with members of any other proposed or established family.

### 2.9 Functional annotation of jumbo phage genomes

We performed comprehensive functional annotation through sequential analytical approaches. Proteins were initially annotated against Pfam-A, TIGRFAM, and VOGDB databases using hmmsearch (-E 1e-5). To address unannotated proteins, we conducted specialized blastp analyses against jumbo phage terL and major capsid protein databases (--min-score 50 -e 1e-5).

Antibiotic resistance genes were identified using Resistance Gene Identifier v5.1.0 (45) with gene-specific bit-score thresholds. Potential virulence factors were detected via BLASTp alignment against VFDB_setB_pro.fas (46), requiring ≥60% identity and ≥70% query coverage for annotation.

Auxiliary metabolic genes (AMGs) underwent dual detection using VIBRANT v1.2.1 (47) and DRAM-v v1.3.5 (48). Proteins were first scored by VirSorter2 v2.2.2 (25), then annotated through DRAM-v with default settings. AMGs were pathway-mapped using KEGG (49) and retained only when corroborated by both VIBRANT and DRAM-v.

### 2.10 Estimation of abundances for novel family jumbo phages

We quantified jumbo phage abundances by mapping quality-filtered metagenomic reads from PGV and seven pig gut microbiome studies to jumbo phage genomes using BWA MEM v0.7.17 (50) with default parameters. Resulting alignments were converted to BAM format using Samtools v1.15 (51). Average coverage depths per genome per sample were calculated via Bedtools v2.30.0 (52) genomecov function.

Genome abundances were set to zero when sample reads provided <1 × coverage across ≥75% of the genome. Final abundances for novel family jumbo phages were subsequently computed. Additionally, we performed FastSpar correlation analysis between jumbo phages and novel jumbo phages based on abundance profiles. Correlation significance was assessed using a Bootstrap-based procedure with 1,000 iterations, and only statistically significant relationships (*p* < 0.001) were retained for downstream analysis.

### 2.11 Statistical analyses

All statistical analyses were conducted using R v4.2.1. Heatmap visualizations were generated using ComplexHeatmap v2.13.2 (53).

## 3 Results

### 3.1 Recovering jumbo phage genomes from 450 pig gut metagenomes

**Figure 1:**
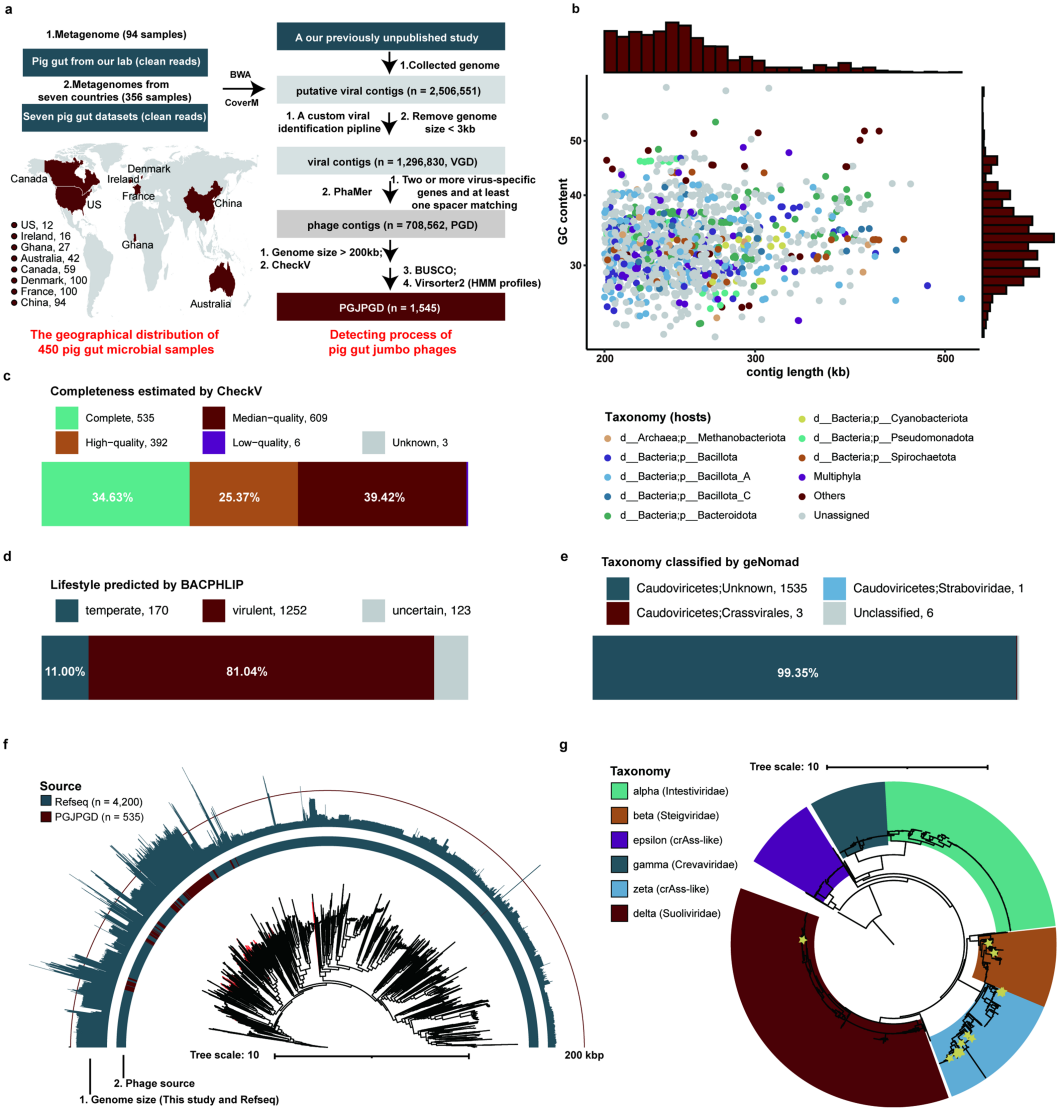
Identification and characterization of jumbo phage genomes from pig gut. **(a)** The geographical distribution of pig metagenomic sequencing data used in this study, and the pipeline for detecting pig gut jumbo phage genomes. **(b)** Genome size and GC content of jumbo phage genomes (*n* = 1,545) reconstructed from the pig gut. Bar plots on the top and right side depict the distribution of genome size and GC content, respectively. **(c–e)** The completeness **(c)**, lifestyle prediction **(d)**, and taxonomy **(e)** of pig gut jumbo phage genomes. **(f)** The phylogenetic tree analysis of *Caudoviricetes* genomes based on complete genomes of jumbo phages recovered in this study and phage genomes from the Refseq. The genome sizes are shown in the outer cycle, and the source of genomes are indicated in the inner cycle. **(g)** The phylogenetic tree analysis of crAss-like phages using genomes of pig gut jumbo phages and crAss-like phages reported previously.

We established a custom pipeline (Figure 1a) to recover jumbo phages from 450 pig gut metagenomes across eight countries (Supplementary Table S1), identifying 1,545 jumbo phage genomes. Host prediction revealed 53 generalist phages (3.43%) targeting multiple phyla, primarily infecting key pig gut phyla Bacillota_A and Bacteroidota. Notably, 16 archaea-infecting jumbo phages (1.04%) targeted Methanobacteriota (Figure 1b). Genome quality assessment showed 1,536 jumbo phages (99.42%) with ≥50% completeness, while lifestyle prediction classified 1,252 as virulent (Figures 1c,d). Taxonomic annotation placed 1,539 genomes (99.61%) in Caudoviricetes, three in Crassvirales, and one in Straboviridae, with six (0.26%) unclassified at family level (Figure 1e; Supplementary Table S2).

We analyzed 535 complete Caudoviricetes genomes alongside 4200 RefSeq references using 77 marker genes. This expanded Caudoviricetes diversity and revealed jumbo phages’ close phylogenetic relationships, supporting their genomic stability hypothesis (Figure 1f). Crucially, we identified 23 crAss-like jumbo phages (>200 kb) – a previously unreported size category in pigs (Figure 1g). These findings address mammalian gut jumbo phage research gaps while highlighting genomic novelty.

### 3.2 Characterization of potential competitive networks among pig gut jumbo phages via CRISPR spacers

**Figure 2:**
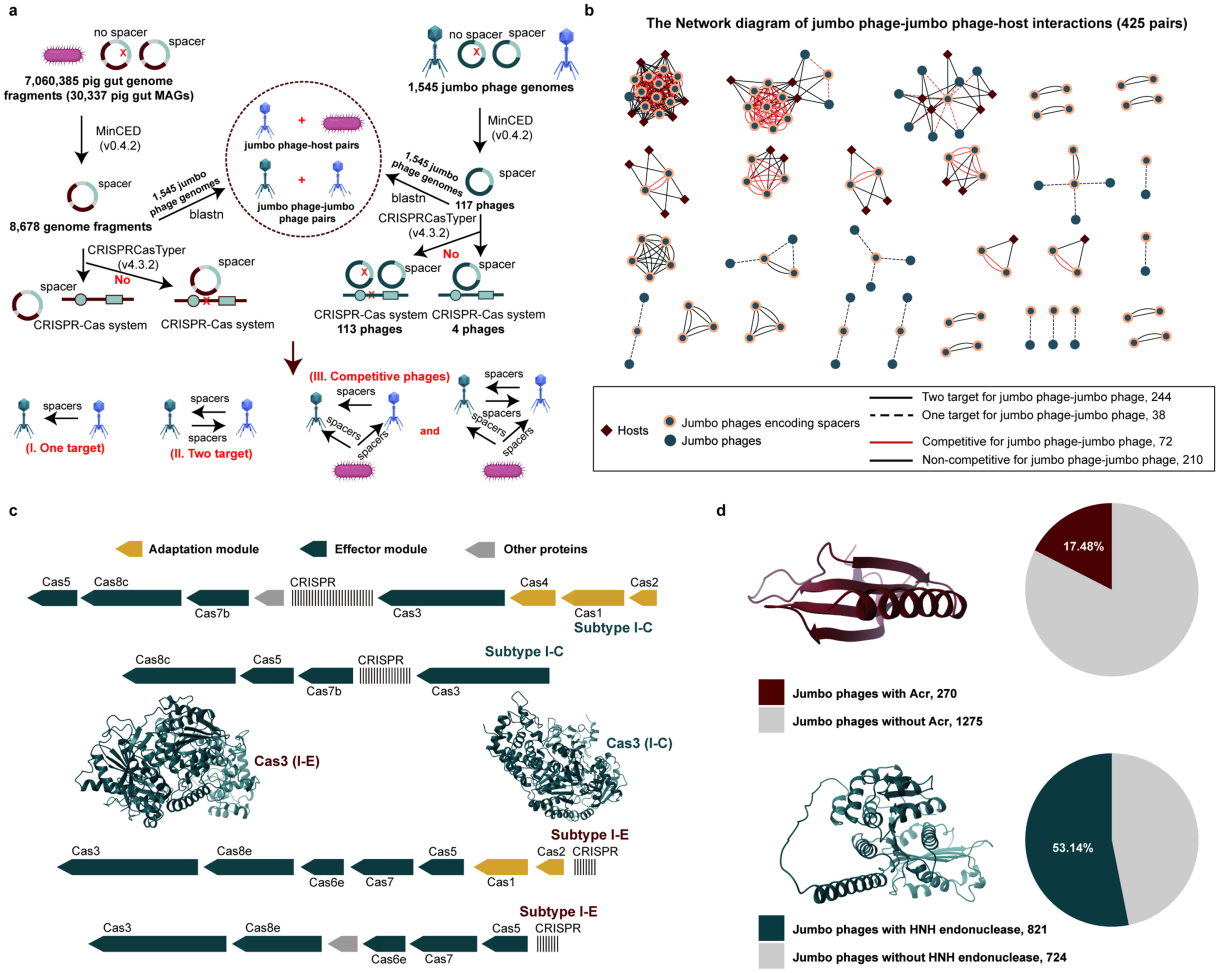
Revealing the interactions between jumbo phages, and between jumbo phages and hosts. **(a)** Identification of jumbo phages and hosts with CRISPR spacers and CRISPR-Cas systems. **(b)** The interaction networks among jumbo phages. Dots represent different jumbo phages, and diamonds represent hosts. The line colors represent competitive or non-competitive phages, and the solid and dot lines represent one-target pairs and two-target pairs. **(c)** CRISPR-Cas systems in jumbo phage genomes and 3D structure of the Cas3 proteins from different subtypes of CRISPR-Cas systems. **(d)** The proportion of pig gut jumbo phages harboring genes encoding Acr and HNH endonuclease proteins.

Despite documented CRISPR-Cas systems in jumbo phages protecting hosts against competing phages (5, 7), potential competitive networks remain poorly characterized. Our analysis of pig gut prokaryotic and jumbo phage genomes revealed a paradox: while numerous genomes contained CRISPR spacers, complete CRISPR-Cas systems were exceptionally rare (Figure 2a).

We reconstructed potential competitive networks through CRISPR spacer matching, identifying 282 jumbo phage-jumbo phage interactions and 143 jumbo phage-host interactions (Figure 2b). Strikingly, 86.52% (*n* = 244) of phage-phage interactions exhibited mutual targeting patterns, with 16.94% (*n* = 72) confirmed as competitive relationships.

Focusing on the four jumbo phages with complete CRISPR-Cas systems (Figure 2c), we identified subtype I-C and I-E systems. Complementary analysis revealed 270 jumbo phages encoding anti-CRISPR proteins (Acrs) and 821 encoding HNH endonucleases, defensive countermeasures facilitating host infection (Figure 2d).

Collectively, these findings demonstrated dual defensive strategies: jumbo phages might both protect their hosts against competitors through CRISPR systems while might employing Acrs and HNH endonucleases to overcome host defenses during infection.

### 3.3 Identification of novel phage families through gene-sharing network analysis

**Figure 3:**
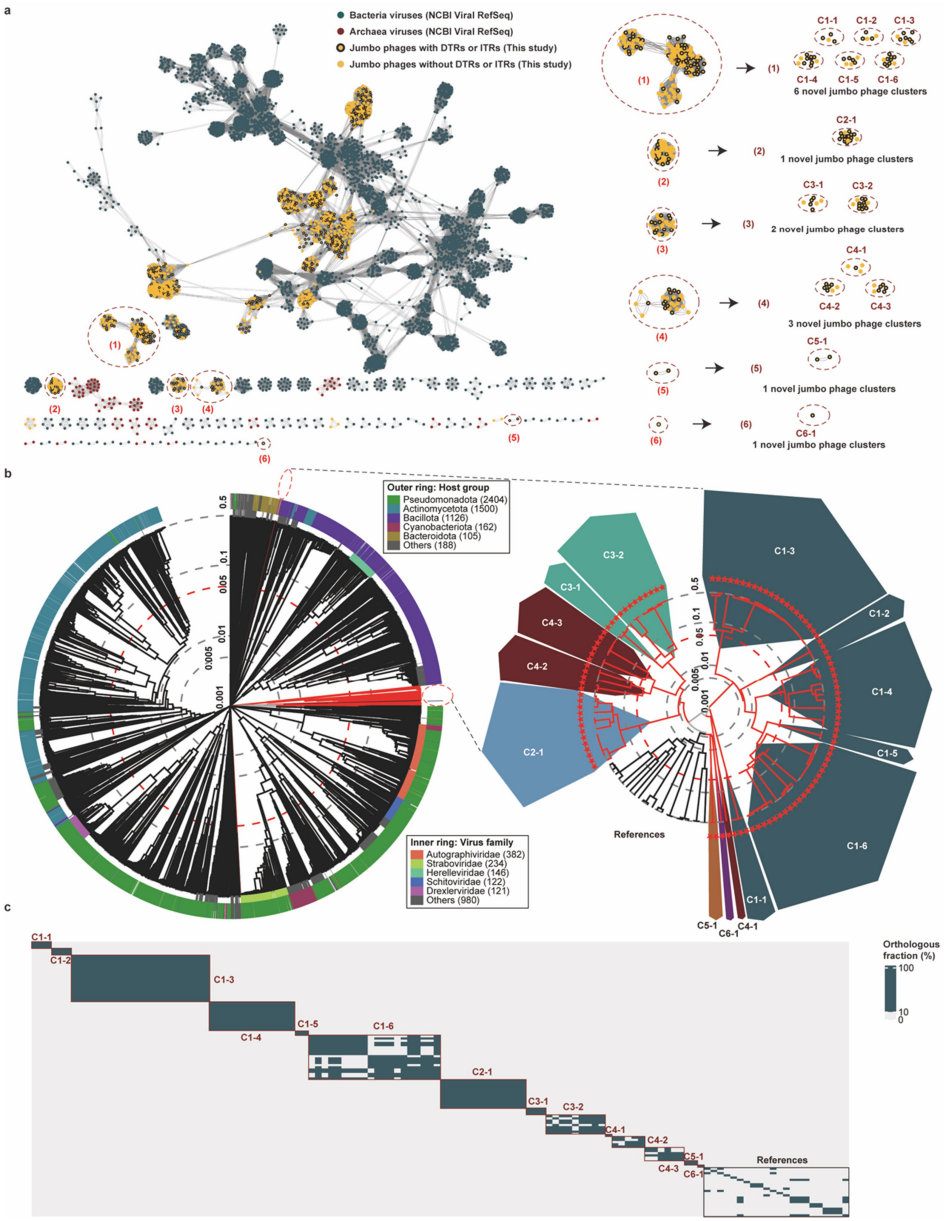
Identifying and validating novel phage families from pig gut jumbo phage genomes. **(a)** Gene-sharing network analysis of pig gut jumbo phage genomes and prokaryotic viral genomes from the RefSeq. Each node represents a viral genome, and each edge denotes the existence of shared proteins among clusters. The categories of viruses are distinguished by colored dots. Bacterial and archaeal viral genomes were retrieved from the RefSeq (release 211). **(b)** Whole proteome-based phylogeny for the taxonomic proposal of novel pig gut jumbo phages. The whole proteome-based phylogenetic tree (left) was constructed from complete jumbo phages and reference viruses using the ViPTree pipeline (https://www.genome.jp/viptree/); note that this method did not generate bootstrap support values. For clarity, a curated subtree (right) is shown, comprising all complete jumbo phages and their closest reference viruses extracted from the full tree. Pig gut jumbo phages with complete genomes are indicated by red lines and reference viral genomes are shown in black line and font. Red dashed lines in the log-scale phylogenetic trees denote the branch length demarcation for family-level taxonomies (~0.05). **(c)** Percentage of shared orthologous proteins by pairwise jumbo phage genomes for the taxonomic proposal of novel pig gut jumbo phages. ≥10% of shared orthologous proteins are marked by dark green fill, and jumbo phage genomes from the same family and reference are denoted by the boxes with black borders on the heatmaps.

To comprehensively assess pig gut jumbo phage diversity, we performed gene-sharing network analysis integrating 1,545 genomes with RefSeq prokaryotic viruses. Notably, 236 jumbo phage genomes formed isolated network nodes without RefSeq connections, significantly expanding known viral diversity (Figure 3a). Six genome clusters containing complete representatives showed no RefSeq affiliations, representing uncharacterized virosphere taxa.

Proteome-based phylogenetic trees of these clusters with reference viruses revealed deep branching (Figures 3b,c; Supplementary Table S3). According to the established criteria (43, 54, 55), the branch length for family-level viral assignment in the proteome-based phylogeny is ~0.05, and viruses with <10% of shared orthologous proteins belong to different viral families. We proposed 14 novel viral families designated: C1-1 (*n* = 15), C1-2 (*n* = 4), C1-3 (*n* = 35), C1-4 (*n* = 36), C1-5 (*n* = 2), C1-6 (*n* = 51), C2-1 (*n* = 34), C3-1 (*n* = 12), C3-2 (*n* = 19), C4-1 (*n* = 3), C4-2 (*n* = 12), C4-3 (*n* = 10), C5-1 (*n* = 2), and C6-1 (*n* = 1).

### 3.4 Functional potentials and host associations of putative novel pig gut jumbo phage families

**Figure 4:**
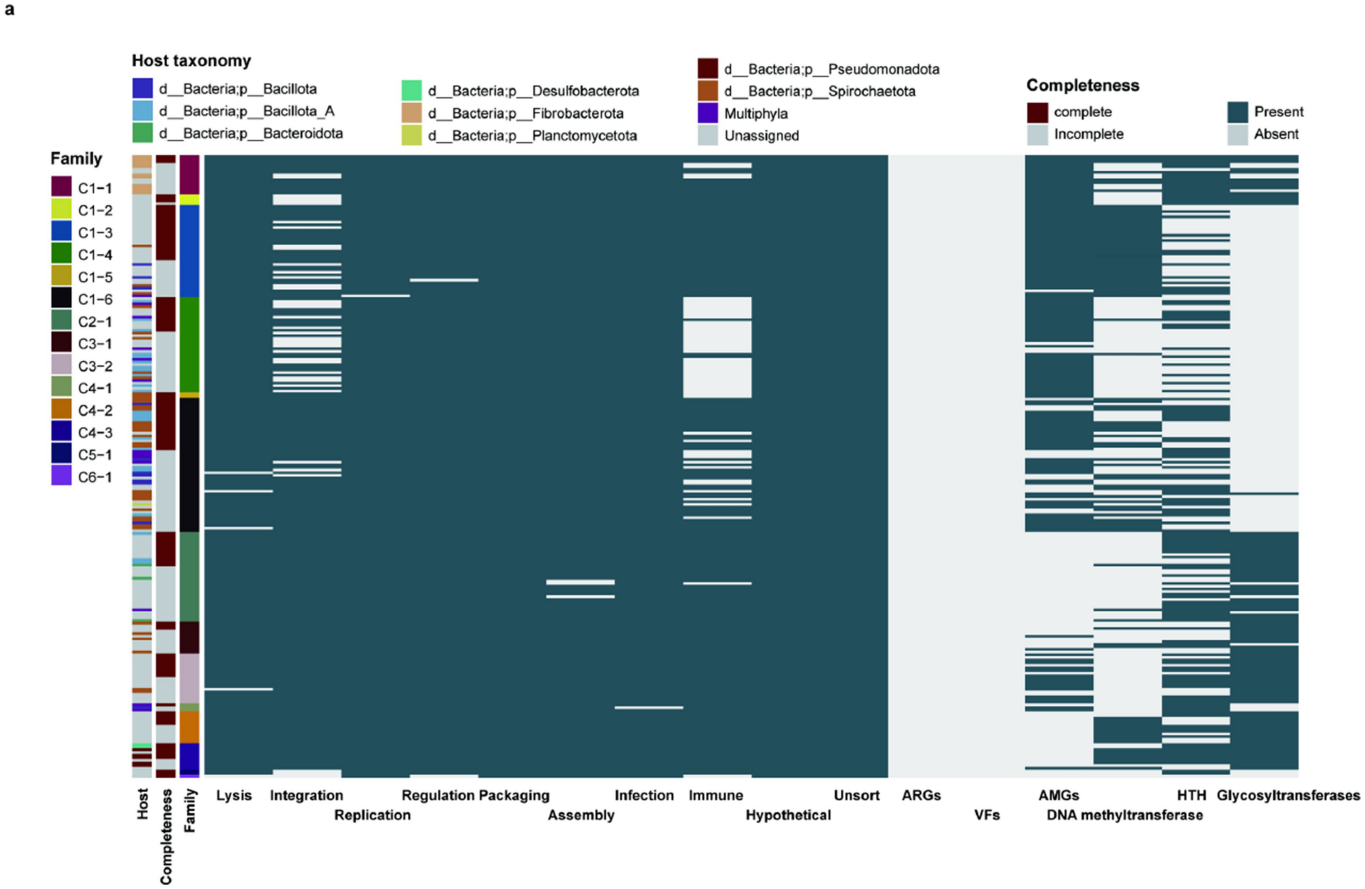
Functional potentials of novel jumbo phage families. Function and host of jumbo phages (*n* = 236) from novel archaeal viral families. The first three colored strips indicate host bacteria at the phylum level, genome completeness, and novel families. The dark green blocks on the heatmap represent the presence of function items for jumbo phage genomes from novel phage families.

Functional analysis of 236 genomes representing 14 novel jumbo phage families revealed significant knowledge gaps: most genes were annotated as hypothetical or unclassified (Figure 4a; Supplementary Table S4). Among functionally resolved genes, we identified key roles in lysis, integration, replication, regulation, packaging, assembly, infection, and immune processes.

Strikingly, while no antibiotic resistance or virulence genes were detected, auxiliary metabolic genes (AMGs) appeared in 10 families (C1-1, C1-2, C1-3, C1-4, C1-5, C1-6, C3-1, C3-2, C4-1, C4-3), suggesting metabolic modulation of hosts. Notably, 12 families (C1-1, C1-2, C1-3, C1-4, C1-6, C2-1, C3-1, C3-2, C4-1, C4-2, C4-3, C5-1) encoded defense-evasion systems including DNA methyltransferases, HNH endonucleases, and glycosyltransferases known to circumvent host immunity.

Host association analysis revealed family-specific patterns: C1-1 targeted Fibrobacterota, while C1-5, C3-1, and C3-2 associated with Spirochaetota. The remaining ten families showed broader tropism, infecting hosts across multiple phyla or unclassified bacteria.

Collectively, these findings demonstrate that novel jumbo phage families possess diverse functional repertoires enabling both metabolic manipulation and sophisticated host immune evasion.

### 3.5 Global prevalence and co-abundance networks of putative novel pig gut jumbo phage families

**Figure 5:**
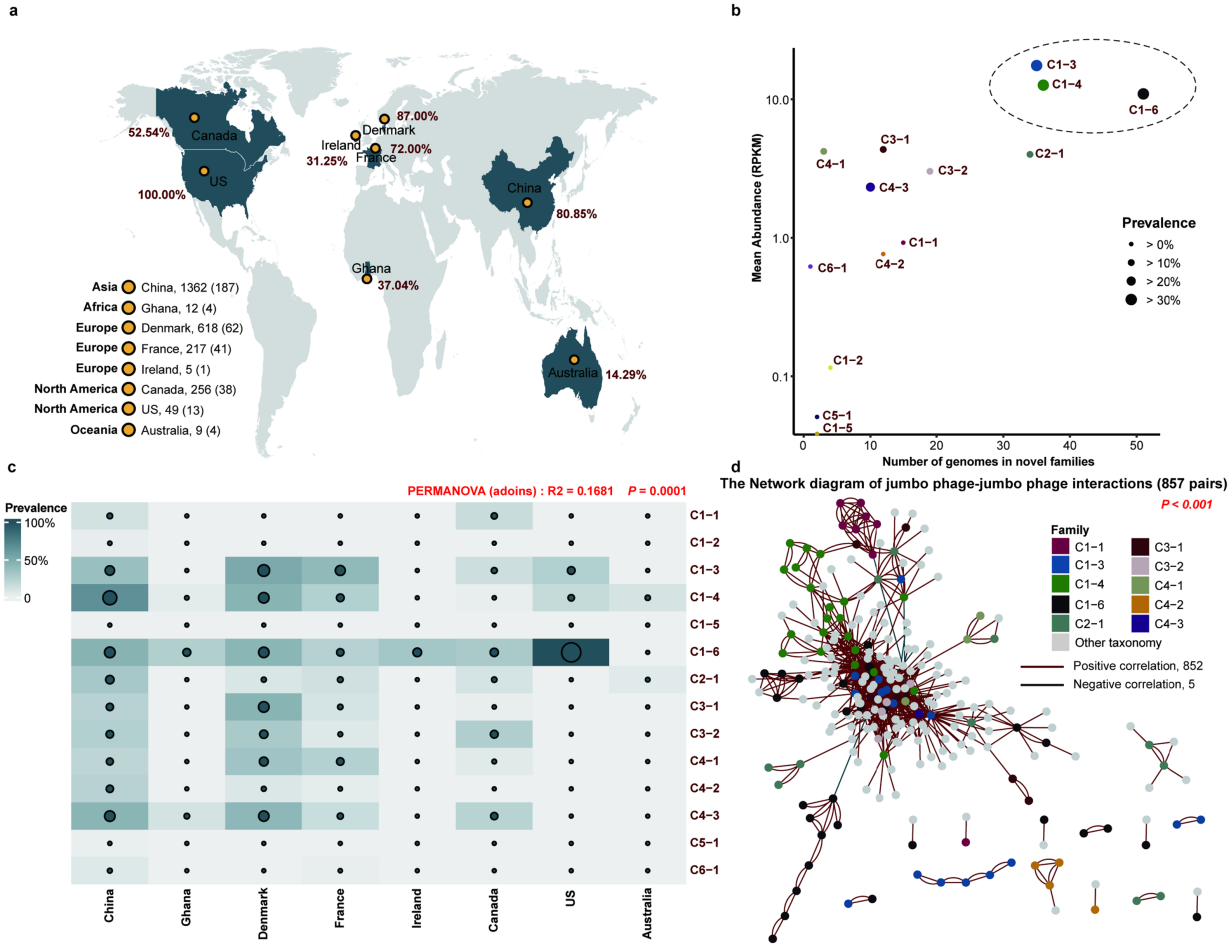
Prevalence of novel jumbo phage families and co-abundance network of jumbo phages. **(a)** Geographic distribution of jumbo phage genomes from novel families. **(b)** Number of jumbo phage genomes and average abundance for each novel family. Different novel families of jumbo phages are indicated by different colors. **(c)** Prevalence of novel jumbo phage families in different countries. **(d)** The co-abundance network (*p* < 0.001) of jumbo phages from the pig gut. The dots represent jumbo phage genomes, and the size of the dots represents the different prevalence of jumbo phage genomes in the pig gut. Different novel families of jumbo phages are shown by different colors. The red lines and black lines represent the positive correlations and negative correlations, respectively.

Our global analysis across eight countries revealed that all studied regions harbored at least one representative from the 14 novel jumbo phage families, with China exhibiting the highest diversity of unique jumbo phages (Figure 5a; Supplementary Tables S5). Among these families, C1-3, C1-4, and C1-6 demonstrated particularly high prevalence (30.22–33.11% of samples) and abundance (Figure 5b). Notable geographical patterns emerged where C1-3 dominated 51.00% of Danish samples, C1-4 appeared in 65.96% of Chinese samples, and C1-6 was ubiquitous across all US samples (Figure 5c), indicating significant inter-country distribution heterogeneity.

Co-abundance network analysis of these novel families identified 852 positive correlations versus only five negative interactions (Figure 5d). Genomes within the same family consistently showed stronger co-abundance relationships than those between different families. These findings collectively highlight the ecological significance of novel jumbo phage families in maintaining pig gut ecosystem stability.

## 4 Discussion

While gut microbiome research has predominantly centered on bacteria, the role of jumbo phages remains underexplored. Our study provides a substantial advance by identifying 1,545 jumbo phage genomes from the pig gut and proposing 14 potential novel families (43, 56), thereby significantly expanding the known diversity of these large phages. This resource is characterized by several key novelties. First, phylogenomic analyses revealed substantial unexplored diversity within the class Caudoviricetes. Second, these novel families exhibit distinct geographical prevalence patterns (e.g., families C1-3, C1-4, and C1-6 being enriched in Denmark, China, and the US, respectively), supported by significant PERMANOVA results (R^2^ = 0.1681, *p* = 0.0001), suggesting habitat-specific adaptation. Third, beyond taxonomic novelty, these families encode unique functional repertoires. Notably, 9 families are enriched with auxiliary metabolic genes (AMGs) and sophisticated anti-host defense systems (e.g., DNA methyltransferases, HNH endonucleases) (57), indicating a heightened capacity for host manipulation and participation in co-evolutionary arms races (58). Some families, like C1-6, further deviate by lacking typical regulation and integration modules, hinting at unconventional life-cycle strategies.

The large genomes of jumbo phages appear to facilitate a complex and distinct ecological role. We found evidence of a multifaceted interaction network, including potential competition between jumbo phages and other jumbo phages (inferred from CRISPR spacers and negative co-abundance correlations) and intricate, often defense-oriented relationships with bacterial hosts. This is underscored by the prevalence of the aforementioned AMGs and defense systems within the novel families, suggesting their interactions extend beyond simple predation to potentially modulate host metabolism and community dynamics in ways (59, 60).

Despite these insights, several critical questions require further research. The metagenomic approach, while powerful for discovery, limits definitive host assignment and functional validation. Future work must prioritize isolating key jumbo phages alongside their bacterial hosts to experimentally verify their life cycles, the activity of predicted AMGs, and the ecological impact of their defense systems (1, 61). Furthermore, the causal mechanisms behind the observed geographical associations and the precise ecological consequences of phage-phage competition networks remain to be elucidated. *In vitro* and *in vivo* models are needed to test how these jumbo phages influence gut microbiome stability and host health.

In conclusion, our study establishes a foundational genomic resource that redefines the pig gut virome, highlighting jumbo phages as diverse entities capable of complex interactions through their expanded genetic toolkit. This work shifts the paradigm from viewing phages solely as predators to recognizing them as potential modulators of ecosystem function, paving the way for future applications in phage therapy (62, 63) and microbiome engineering.

## 5 Conclusion

In conclusion, this work identified 1,545 jumbo phage genomes from 450 pig gut metagenomes. Using CRISPR spacer analysis, we predicted archaeal or bacterial hosts and reconstructed potential competitive phage networks within this ecosystem. We expand genomic resources for pig gut viromes and delivers novel insights into jumbo phage functional capabilities, and host associations.

## Data Availability

All pig gut jumbo genomes associated in the study can be accessed and downloaded without any restriction at https://doi.org/10.5281/zenodo.16780294. The codes for identification of jumbo phage https://doi.org/10.5281/zenodo.16780294.

## Footnotes

1
http://vogdb.org

2
https://itol.embl.de/

3
https://www.genome.jp/viptree

4
https://github.com/dparks1134/CompareM
